# Characterization and Classification of Spanish Honey by Non-Targeted LC–HRMS (Orbitrap) Fingerprinting and Multivariate Chemometric Methods

**DOI:** 10.3390/molecules27238357

**Published:** 2022-11-30

**Authors:** Víctor García-Seval, Javier Saurina, Sònia Sentellas, Oscar Núñez

**Affiliations:** 1 Department of Chemical Engineering and Analytical Chemistry, University of Barcelona, Martí i Franquès 1-11, E08028 Barcelona, Spain; 2 Research Institute in Food Nutrition and Food Safety, University of Barcelona, Recinte Torribera, Av. Prat de la Riba 171, Edifici de Recerca (Gaudí), Santa Coloma de Gramenet, E08921 Barcelona, Spain; 3 Serra Húnter Fellow, Generalitat de Catalunya, Via Laietana 2, E-08003 Barcelona, Spain

**Keywords:** blossom honeys, honeydew honeys, LC–HRMS, fingerprinting, chemometrics

## Abstract

A non-targeted LC–HRMS fingerprinting methodology based on a C18 reversed-phase mode under universal gradient elution using an Orbitrap mass analyzer was developed to characterize and classify Spanish honey samples. A simple sample treatment consisting of honey dissolution with water and a 1:1 dilution with methanol was proposed. A total of 136 honey samples belonging to different blossom and honeydew honeys from different botanical varieties produced in different Spanish geographical regions were analyzed. The obtained LC–HRMS fingerprints were employed as sample chemical descriptors for honey pattern recognition by principal component analysis (PCA) and partial least squares–discriminant analysis (PLS–DA). The results demonstrated a superior honey classification and discrimination capability with respect to previous non-targeted HPLC–UV fingerprinting approaches, with them being able to discriminate and authenticate the honey samples according to their botanical origins. Overall, noteworthy cross-validation multiclass predictions were accomplished with sensitivity and specificity values higher than 96.2%, except for orange/lemon blossom (BL) and rosemary (RO) blossom-honeys. The proposed methodology was also able to classify and authenticate the climatic geographical production region of the analyzed honey samples, with cross-validation sensitivity and specificity values higher than 87.1% and classification errors below 10.5%.

## 1. Introduction

Honey is a natural sweet substance produced by *Apis mellifera* bees from the nectar of plant flowers (known as blossom honey or nectar honey) or from the excretions of plant-sucking insects (*Hemiptera*) on the living part of plants or the secretions of living parts of plants (known as honeydew honeys). The bees collect the nectar, plant secretions or insect excretions and transform them by combining them with specific substances of their own. The generated substance is deposited, dehydrated, stored and kept in honeycombs to ripen and mature into honey [1,2]. Honeys can also be classified regarding their botanical variety origin. When a specific botanical variety prevails, maintaining the physicochemical, microscopic and organoleptic characteristics of that source, honey is considered as monofloral and may then be marketed under the name of the predominant botanical variety. In contrast, honey is considered multifloral (or polyfloral) when comes from a diversity of botanical sources. Although there are requests for the European Commission to amend the Honey Directive [1,2] with a view to providing clear definitions and setting out the main distinctive characteristics of apiculture products, such as monofloral against multifloral honeys [3], there are still discrepancies regarding the minimum content from a certain botanical source required to consider a honey as monofloral. Indeed, the established levels, based on the pollen percentages, depend on each national country’s legislation, provisions, decisions or guidelines [4]. In any case, most of these national criteria have established 45% as the minimum pollen percentage of a given botanical source for the honey to be considered monofloral [4]. Nevertheless, these percentages can change considerably depending on the country and the botanical source; for example, values higher than 85–90% (depending on the country) for chestnut honey, higher than 85% (in Germany) for eucalyptus honey or down to 3–20% (depending on the country) for citrus honey have been established [4]. 

Honey is a highly appreciated product consumed since ancient times mainly due to its health benefits, it being a good source of antioxidants with anti-inflammatory, antibacterial and antifungal properties [5,6,7]. Other well-known health benefits of honey comprise its wound healing capacity, sore throat and cough soothing ability, help in digestive issues and the brain benefits it presents [6]. Although honey is mainly composed of sugars (about 76%), mainly fructose, glucose and sucrose, and water (18%), it is also a remarkable source of vitamins (such as ascorbic acid, pantothenic acid, niacin and riboflavin), minerals (such as calcium, magnesium, copper, iron, phosphorus, potassium, manganese and zinc) and other bioactive substances such as polyphenolic compounds, the latter being the main substances responsible for their antioxidant properties [8,9,10]. In addition, the presence of these minor components and bioactive substances can be exploited to characterize and discriminate the different honey botanical varieties, especially blossom and honeydew honeys [11,12,13].

Considering that the honey supply is lower than the demand, honey is found among the food products most adulterated in order to obtain a fraudulent economic benefit, for example, from the addition of other sugar-based adulterants or syrups [14]. This can also lead to adverse health effects caused by an increase in blood sugar, which can cause diabetes, among other problems [15]. In addition, the great differences in properties and health benefits depending on the honey composition make monofloral honeys the most appreciated and demanded ones. Hence, the assessment of their botanical variety as well as the geographical origin become important authenticity issues, especially for those products with a protected designation of origin (PDO) because of the higher prices that they can reach. The botanical origin of honey is typically determined by the microscopic identification of pollen types (melissopalynological analyses) [16,17], but this method is time-consuming. Within this context, the development of fast, simple and feasible methodologies for honey authentication is required. 

Among the chemical-based methodologies, non-targeted fingerprinting metabolomic strategies in combination with multivariate chemometric methods are gaining popularity to address food authentication issues [18,19]. In contrast to target methodologies, which are focused on the determination of specific chemical metabolites (i.e., sample bioactive markers indicative of a certain property of the food product), non-targeted fingerprinting does not deal with the identification of metabolites but on the recognition of patterns, the so-called fingerprints [20]. Thus, non-targeted fingerprinting strategies deal with registering as much chemical information as possible from the analyzed samples (by means of high-throughput screening methodologies), aiming to differentiate and classify the analyzed samples from the establishment of sample patterns [21]. Recording spectral information by ultraviolet–visible (UV–vis), fluorescence (FL) or near-infrared (NIR) fingerprinting spectroscopies is widely employed for honey pattern recognition to classify and authenticate honey samples according to their botanical or geographical origins and to prevent fraudulent practices [22,23,24,25,26]. Liquid chromatography (LC) with UV–vis detection, often in combination with low-resolution (LC–MS) or with high-resolution mass spectrometry (LC–HRMS), has also been employed to address honey authentication issues by means of non-targeted fingerprinting [27,28,29,30].

In a previous study, we developed a non-targeted HPLC–UV fingerprinting method by using honey dilution with water and methanol as a simple sample treatment procedure, which provided an acceptable discrimination between blossom and honeydew honeys as well as among several blossom honey botanical varieties [30]. Nevertheless, no discrimination at all was accomplished between the honeydew honey botanical varieties nor regarding the honey geographical origins. The present work aimed to develop a non-targeted LC–HRMS chromatographic fingerprinting methodology for the characterization, classification and authentication of Spanish honey samples based on both their botanical and geographical origins. Multivariate chemometric methods such as principal component analysis (PCA) and partial least squares–discriminant analysis (PLS–DA) were employed to assess honey classification and authentication using non-targeted LC–HRMS fingerprints as the source of information.

## 2. Results and Discussion

### 2.1. Non-Targeted LC–HRMS Fingerprints

**Figure 1:**
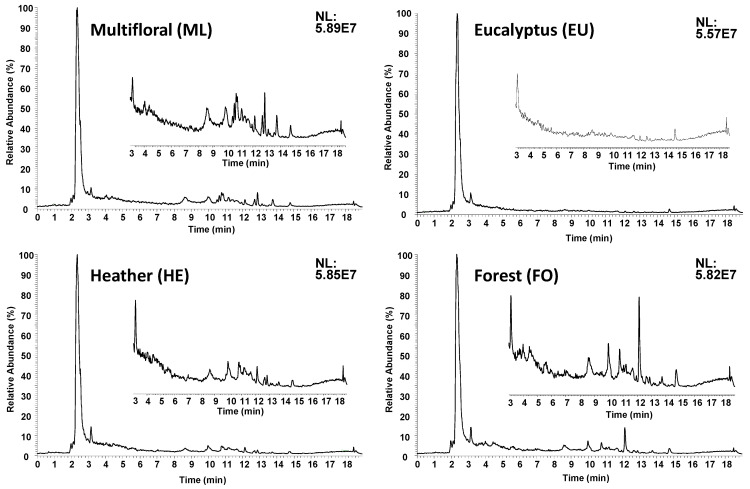
Non-targeted LC–HRMS (total ion chromatogram) fingerprints obtained for multifloral, eucalyptus, heather and forest honeys.

As described in the literature, polyphenolic compounds are among the most important bioactive substances present in honey [10,11,12], reversed-phase LC–MS methodologies in a negative ESI mode being among the most frequently employed conditions for the determination of this family of compounds [31]. In the present contribution, a non-targeted LC–HRMS metabolomic fingerprinting analysis of blossom and honeydew honeys was carried out with the aim of obtaining good chemical descriptors to accomplish sample classification and authentication according to both the botanical variety and geographical production region. A simple sample treatment consisting of dissolving the honey sample with water followed by a 1:1 dilution with methanol, as described in Section 3.2, was employed. The samples were then analyzed by a C18 reversed-phase LC–HRMS method using a universal gradient elution program from 3 to 95% acetonitrile over 15 min. The data were acquired in a full scan HRMS (*m*/*z* 110–1000) at 60,000 full-width at half-maximum (FWHM) resolution and in a negative ESI polarity. The fingerprints obtained for each honey sample depended on both the honey botanical variety genotype and the product phenotype (food attributes that are determined by climatological conditions, among others). Thus, the proposed non-targeted metabolomic fingerprinting strategy relied on obtaining as rich LC–HRMS fingerprints as possible, consisting of ion intensity registered as a function of *m*/*z* values and retention time. As an example, the obtained non-targeted LC–HRMS fingerprints (total ion chromatograms, TIC) of four selected honey samples are depicted in Figure 1.

As can be seen in the figure, the obtained non-targeted LC–HRMS TIC fingerprints were characterized by a huge peak signal corresponding to the column dead volume (around 2.5 min) with a coelution of all the non-retained compounds under reversed-phase mode, these being mainly sugar-related components. Apart from this peak, the total ion chromatograms revealed important differences among the analyzed samples, as can be observed in the figure amplifications from 3 to 18 min. These differences consisted of not only the number of signals detected but also the peak signal intensities. For example, the blossom multifloral (MF) and the honeydew multifloral forest (FO) honey samples displayed a higher number of peak signals throughout the chromatogram (Figure 1). In contrast, the chromatographic fingerprints of the other honey samples seemed to be much simpler, with few peaks and with lower intensities, such as in the case of the blossom eucalyptus (EU) honey. This could be related to the fact of it being a monofloral honey with an enhanced content of a specific botanical variety in comparison to the multifloral honeys. The observed differences between the different honey varieties analyzed were also highlighted in the non-targeted LC–HRMS fingerprints when considering only the base peak chromatograms (Figure S1 in the Supplementary Materials). In any case, the differences observed between the obtained non-targeted LC–HRMS fingerprints for the different honey types under study, and the fact that the fingerprints seemed to be quite reproducible within the same honey botanical origin, suggested that they could be suitable sample chemical descriptors to address honey classification and authentication.

### 2.2. Honey Exploratory Chemometric Analysis by PCA

**Figure 2:**
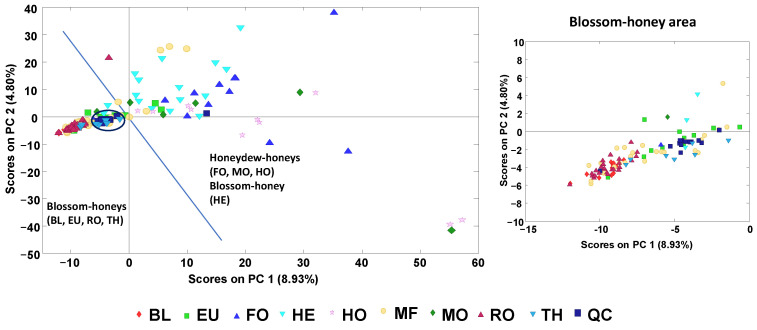
PCA score plot of PC1 vs. PC2 when using non-targeted LC–HRMS fingerprints as honey chemical descriptors. Blue line separates the blossom and honeydew honey (with HE blossom) areas. The blossom honey area is extended on the right plot. BL: orange/lemon blossom; EU: eucalyptus; FO: forest; HE: heather; HO: holm oak; MF: multi-floral; MO: mountain; RO: rosemary; TH: thyme; QC: quality control.

First, the non-targeted LC–HRMS fingerprints were subjected to a non-supervised exploratory method such as PCA to perform a preliminary assessment of honey sample distribution. In addition, the behavior of the quality control (sample analyzed at the beginning of the sequence and once every 10 analyzed samples) was evaluated to ensure the performance of the applied method. As can be seen in the scores plot of PC1 vs. PC2 (Figure 2), the quality controls (QCs) appeared clustered and very close to the center of the plot, demonstrating the reproducibility and robustness of the non-targeted LC–HRMS data and that the chemometric results were not influenced by any sequence drift. 

When addressing the distribution of the analyzed honey samples, two clear behaviors were observed through PC1 related to the honey botanical variety origin. First, a compact group of samples were observed close to the center area of the score plot but exhibiting negative values for both PC1 and PC2. This region was mainly constituted by blossom honeys of the orange/lemon blossom (BL), eucalyptus (EU), rosemary (RO) and thyme (TH) varieties, with the BL and RO varieties being located at more negative PC1 values than the EU and TH ones. Then, a second group of samples were located mainly at positive PC1 values but widely dispersed through PC2, including blossom heather (HE) honey and the honeydew honey samples of the holm oak (HO), forest (FO) and mountain (MO) varieties. These results were similar to those previously reported when using HPLC–UV fingerprints [30], although the blossom honeys here were much more clustered. With both methodologies, the heather samples appeared distributed in the same area as the honeydew honey samples despite being a blossom honey, which was due to their compositional similarities (mainly attributed to phenolic compounds), their similar antioxidant capacity (in general higher than that exhibited by blossom honeys) and their similar color attributes (being normally darker honeys in comparison to blossom ones). Regarding the multifloral (MF) samples, they were dispersed throughout both groups (although they tended to be more concentrated in the blossom honey sample area). This large variability was expected because of their higher diversity of botanical origins.

### 2.3. Classification of Honey Samples by Supervised PLS–DA

**Figure 3:**
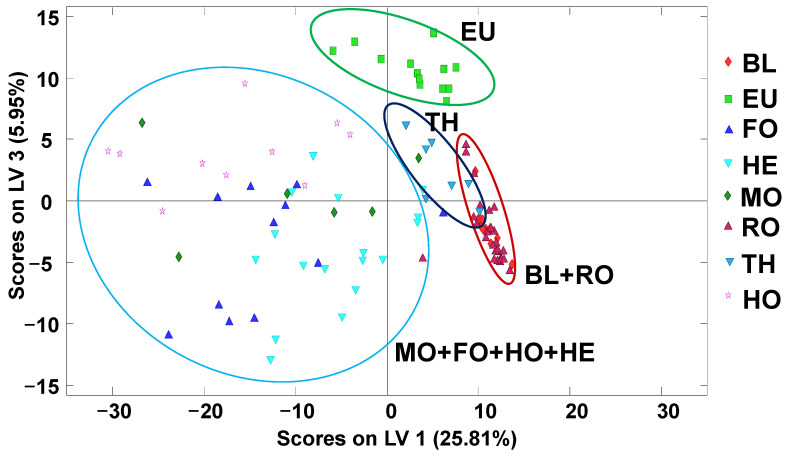
Score plot of LV1 vs. LV3 by PLS–DA when using non-targeted LC–HRMS fingerprints as honey chemical descriptors for botanical variety classification (three LVs were used to build the model). BL: orange/lemon blossom; EU: eucalyptus; FO: forest; HE: heather; HO: holm oak; MO: mountain; RO: rosemary; TH: thyme. QCs and multifloral honeys were not considered.

A supervised and classificatory chemometric method such as PLS–DA was employed to evaluate the obtained non-targeted LC–HRMS fingerprints as honey chemical descriptors of their botanical variety. Figure 3 depicts the PLS–DA score plot of LV1 vs. LV3 (multifloral and QCs were not considered to build the model). These first latent variables captured a low percentage of the total variance of the data matrix due to the complexity of the chromatographic fingerprints. However, despite the quite low total X variance retained, as shown above, the PLS–DA model was able to satisfactorily predict the honey classes. As expected, the results clearly improved on those previously observed by PCA. Two main areas were distinguished across LV1, corresponding to blossom honey (EU, TH, BL and RO) samples (positive LV1 values) and honeydew honey and HE honeys (negative LV1 values). In addition, although the samples were widely distributed through LV3, a certain discrimination could be observed for some varieties; for example, almost all the HO samples were located at positive LV3 values, and most of the HE samples were located at negative ones. Overall, a much better sample discrimination was observed, which clearly improved on the results previously reported when using HPLC–UV fingerprints and PLS–DA [30]. Eucalyptus blossom honeys were separated from the other classes at the top of the plot. Another cluster of samples, constituted by BL and RO varieties, was observed on the right, and, finally, the thyme samples were grouped in the middle between the BL+RO and the MO+FO+HO+HE groups. The discrimination between the blossom honey samples was probably due to differences in their flavonoid composition according to the literature [32]. In summary, the proposed non-targeted LC–HRMS fingerprinting methodology exhibited a higher classification capability according to the honey botanical varieties than the ones previously reported by non-targeted HPLC–UV fingerprinting (where the same sample treatment was employed) and by off-line SPE HPLC–UV polyphenolic fingerprinting (based on a specific polyphenolic isolation sample treatment employing HLB cartridges) [30].

**Figure 4:**
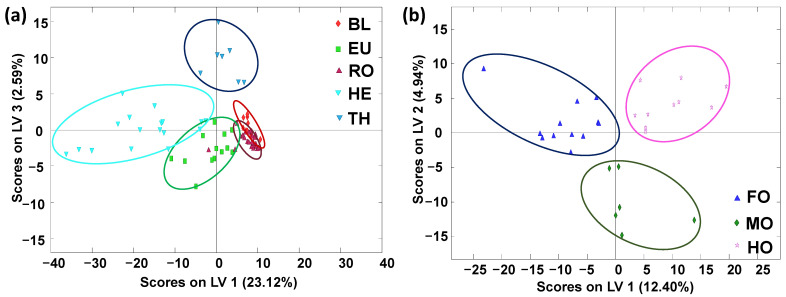
PLS–DA score plots when using non-targeted LC–HRMS fingerprints as honey chemical descriptors for (**a**) blossom honey botanical variety classification (LV1 vs. LV3 score plot, three LVs were used to build the model) and (**b**) honeydew honey botanical variety classification (LV1 vs. LV2 score plot, three LVs were used to build the model). BL: orange/lemon blossom; EU: eucalyptus; FO: forest; HE: heather; HO: holm oak; MO: mountain; RO: rosemary; TH: thyme.

The classification of the analyzed honey samples based on their botanical variety origin was also evaluated by PLS–DA by employing blossom honey and honeydew honey subsets independently. As can be seen in the PLS–DA score plots, an excellent sample discrimination was obtained in both cases (Figure 4). The samples tended to be clustered in five groups when studying all the blossom-honey samples (LV1 vs. LV3 PLS–DA score plot) with some overlapping between HE and EU and between EU, RO and BL. The thyme (TH) samples were clearly discriminated at the top of the plot (Figure 4a). Perfect discrimination was accomplished when the paired PLS–DA models were considered, as can be seen in Figure S2 (supplementary materials) for the PLS–DA plot of BL vs. RO. Regarding the honeydew honey subset, the samples were clustered into three groups with no overlapping (see Figure 4b). This level of separation was not accomplished with any of the previously reported HPLC–UV fingerprinting methodologies [30], where, for example, the honeydew honey samples were always overlapped. This finding demonstrated again the higher classification capabilities of the developed non-targeted LC–HRMS fingerprinting method.

Multiclass PLS–DA models were built for specifically working with blossom honey (including BL, RO, EU, TH and RO classes) and honeydew honey (including HO, MO and FO classes) subsets independently. Table 1 summarizes the sensitivity, specificity and overall classification prediction error assessed by cross-validation for the blossom honey samples. The sensitivity and specificity values were higher than 96.2%, except for the specificity for BL and RO (57.1% and 79.6%, respectively), them being the two groups with higher overlapping. Accordingly, the classification error of these two groups also worsened, with values higher than 12.7%, while for the other three groups the classification errors were below 1.7%. The PLS–DA classification of the honeydew–honey samples achieved 100% sensitivity and specificity, with 0% prediction error (Table S1, Supplementary Materials). Overall, excellent results were accomplished, and, as previously commented, the classification capabilities of the proposed methodology noticeably improved when paired PLS–DA models of the BL vs. RO samples were employed (Figure S2, Supplementary Materials).

It should be mentioned that, in this work, no melissopalynological analysis of the employed honey samples was performed, and, therefore, the PLS–DA classification studies were based on the botanical origin declared on the honey sample labels. Nevertheless, this was not a disadvantage for this study. First, the minimum percentage of pollen from a given botanical source needed to claim a specific botanical origin is ambiguous [4], as previously commented in the introduction section. For instance, the minimum percentage of pollen to claim that a honey is citrus honey is 3–20% depending on the country. This means that the specific origin for the 80–97% remaining does not need to be declared. It is important to have in mind that different botanical species grow in specific areas depending on the characteristics of the soil, the climate and the water supply as well as agricultural practices. Thus, the botanical diversity in ecosystems that share species should be similar. This means that the characteristics of those monofloral-claimed honey production areas may be similar for a given botanical origin. Hence, although the content of pollen in different honey samples of a specific botanical source can vary among producers, brands or production years, it is reasonable to think that all of them will share the same global characteristics. Indeed, the obtained results showed a reasonable concordance in the fingerprints of samples with similar characteristics, assuming the validity of the PLS–DA classification results as a proof of concept.

The capability of the non-targeted LC–HRMS fingerprints to classify the analyzed honey samples based on their geographical production origin was also evaluated. Similarly to the results previously reported by HPLC–UV fingerprinting [30], no discrimination was obtained when considering the different Spanish geographical production regions under study (indicated in Table S2), which was expected considering that it is very difficult to delimit these geographical regions when addressing bee-produced natural products such as honey. In contrast, when considering bigger geographical regions based on climatic conditions, i.e., the north of Spain (Cantabrian Sea region, CBR), the continental area (landlocked inland region, LIR) and the east/south of Spain (Mediterranean Sea region, MSR), the classification results notably improved. 

**Figure 5:**
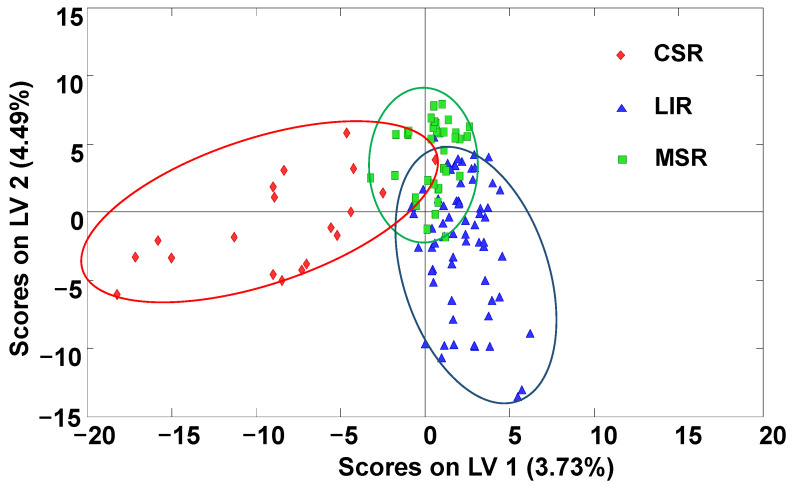
PLS–DA score plots of LV1 vs. LV2 when using non-targeted LC–HRMS fingerprints as chemical descriptors for climatic geographical production region (two LVs were used to build the model). CSR: Cantabrian Sea region; LIR: landlocked inland region; MSR: Mediterranean Sea region.

As can be seen in the PLS–DA score plot of LV1 vs. LV2 depicted in Figure 5, despite certain sample overlapping, the samples tended to be grouped according to the geographical climatic area of honey production. The honeys produced in the Mediterranean Sea region were clustered in the center-top area of the plot, while the honeys produced in the Cantabrian Sea region tended to be located in the left area of the plot, exhibiting negative LV1 values. The honeys produced in the continental regions (landlocked inland region) were located at the right-bottom area of the plot, exhibiting mainly positive LV1 values and negative LV2 values. 

A multiclass PLS–DA model was also built to assign the analyzed honey samples according to their geographical climatic production region, and the results of sensitivity, specificity and overall class prediction errors assessed by cross-validation are summarized in Table 2. Satisfactory results were observed, with sensitivity and specificity values higher than 91.9% and 87.1%, respectively. Classification errors were below 10.5%.

Paired PLS–DA models for the classification of samples based on climatic regions were also built, and the obtained score plots of LV1 vs. LV2 are shown in Figure S3 (Supplementary Materials). As can be seen, the discrimination capability of the proposed methodology improved in all cases (LIR vs. MSR; CSR vs. MSR; and CSR vs. LIR), with minimal sample overlapping. Overall, these results were again much better than the ones previously reported when using HPLC–UV fingerprints [30], enhancing the classification of Spanish honey samples not only according to their different botanical varieties but also their climatic production regions.

## 3. Materials and Methods

### 3.1. Reagents and Chemicals

Acetonitrile (UHPLC supergradient ACS quality) and methanol (Chromasolv^TM^ for HPLC, ≥99.9%) were provided by PanReac AppliChem (Barcelona, Spain). Formic acid (≥98%) was obtained from Sigma-Aldrich (St Louis, MO, USA). Water was purified with an Elix 3 system coupled to a Milli-Q instrument from Millipore Corporation (Bedford, MA, USA). The water was filtered with a 0.22 µm nylon membrane filter integrated into the Milli-Q instrument.

### 3.2. Samples and Sample Treatment

A total of 136 Spanish honey samples, purchased from supermarkets and local markets in Spain, were analyzed. Among them, 32 were labelled as blossom multifloral (MF) honeys, 76 as monofloral blossom honeys of different botanical origins (orange/lemon blossom (BL), rosemary (RO), thyme (TH), eucalyptus (EU), and heather (HE)) and 26 as honeydew honeys including holm oak (HO) and mountain (MO) and forest (FO) honeys. Two heather honeys were donated by Miel de Braña (León, Spain). In addition, the analyzed honey samples were obtained from different Spanish geographical production regions. More information regarding the number of honey samples for each botanical variety and geographical region of production is summarized in Table S2 of the Supplementary Material. 

A simple and non-discriminant honey sample treatment was employed as previously described [30]. Briefly, ca. 1 g of honey was dissolved with 10 mL of Milli-Q water in 15 mL PTFE centrifuge tubes from Serviquimia (Barcelona, Spain) and mixed with a vortex (VibraMix, OVAN, Barcelona, Spain). For the crystallized honey samples, the samples were first melted at 45 °C in a water bath, homogenized and weighed at room temperature. The obtained honey extracts were then centrifuged (3500 g, 5 min) in a Rotina 420 Centrifuge (Hettich, Tuttlingen, Germany) to separate non-soluble particles (i.e., bee bread, pollen and proteins) naturally occurring in honey. The aqueous honey extracts were then diluted with methanol (1:1 ratio). Finally, the samples were filtered through syringe membrane filters (0.45 µm) from FILTER-LAB (Barcelona, Spain) into 2 mL HPLC amber glass injection vials and refrigerated at 4 °C until LC–HRMS analysis.

A quality control (QC) solution was prepared by mixing 50 µL of each honey extract. This solution was then employed to assess the robustness and the repeatability of the non-targeted LC–HRMS fingerprints and to ensure that the chemometric results were not affected by any instrumental drifts.

### 3.3. Non-Targeted LC–HRMS Chromatographic Fingerprinting Method

Non-targeted LC–HRMS honey fingerprints were obtained with a Dionex UHPLC system (Germering, Germany) coupled to an FT–HRMS LTQ Orbitrap instrument from Thermo Fisher Scientific (San Jose, CA, USA). A reversed-phase chromatographic separation (Kinetex^®^ C18 porous shell column of 100 × 4.6 mm I.D., 2.6 µm partially porous particle size; Phenomenex, Torrance, CA, USA) under a universal gradient elution was employed as previously described in [30]. Briefly, 0.1% aqueous formic acid and acetonitrile were used as mobile phase components with the elution gradient shown in Table 3. The injection volume was 5 µL.

An electrospray ionization (ESI) source, set in an off-axis position to minimize contamination and operating in negative ionization mode (capillary voltage of −3.5 kV) was employed. Nitrogen was used for the ESI sheath, auxiliary and sweep gases at flow rates of 50, 20 and 2 a.u. (arbitrary units), respectively. ESI vaporizer and capillary temperatures were kept at 25 °C and 350 °C, respectively. The Orbitrap mass analyzer worked in the full-scan HRMS mode (*m*/*z* range from 110 to 1000) at a mass resolution of 60,000 FWHM (full-width at half-maximum at *m*/*z* 200). The Orbitrap system was tuned and calibrated using commercial calibration solutions for the negative ion mode (Thermo Fisher Scientific). Xcalibur software v 4.1 (Thermo Fisher Scientific) was used to control the LC–HRMS system.

All the honey extract samples were analyzed randomly to prevent and minimize any instrumental drift effect on the built chemometric models. Moreover, a QC solution and an acetonitrile blank were injected at the beginning and after every ten sample injections.

### 3.4. Data Matrix 

First, the LC–HRMS raw data were transformed with the MSConvert free software (ProteoWizard, Palo Alto, CA, USA) into an mzML output format [33,34]. For data simplification, 32 bits as a binary encoding precision and threshold peak filter were employed by establishing the absolute intensity as the threshold type at a value of 10,000 counts. Then, the mzMine 3 software was employed to convert the mzML files into a data matrix containing the non-targeted LC–HRMS fingerprints, which consisted of ion signal intensities arranged as a function of samples and variables in rows and columns, respectively [35]. For this purpose, first, exact mass detection was used to generate mass lists for each acquired sample scan, considering a noise level of 1.0 × 10^5^. The next step was to remove false signals with the FTMS shoulder peak filter by setting a Gaussian peak model function and a mass resolution of 70,000. Then, the automated data analysis pipeline (ADAP) chromatogram builder was employed to join the exact mass signals found in contiguous scans in a sample, by establishing a peak time range, an *m*/*z* tolerance and an intensity threshold of 0.5–19 min, 5 ppm and 1.0 × 10^5^, respectively. Isotopes were then removed by considering that the most representative isotope was the most intense and setting an *m*/*z* tolerance of 5 ppm. The Join Aligner option was then applied to match the masses detected across all analyzed samples, with a mass tolerance of 5 ppm, 80% of weight for *m*/*z*, a retention time tolerance of 0.5 min and 1% of weight for retention time. Finally, the aligned feature list was exported to CSV format for subsequent chemometric analysis. The resulting data matrix contained the peak intensity of each variable—characterized by *m*/*z* and retention time—for each sample. Then, the dimension of the working data matrix (samples + QCs × variables) was 151 × 2084, where the variables consisted of MS peaks recorded in the *m*/*z* range from 110 to 1000 throughout the working chromatographic range from 0.5 to 19.0 min.

### 3.5. Chemometric Data Analysis

The SOLO 8.6 chemometric software from Eigenvector Research (Manson, WA, USA) was employed for exploratory principal component analysis (PCA) and supervised partial least squares–discriminant analysis (PLS–DA). More information about the theoretical aspects of the employed chemometric procedures can be found in [36].

The obtained fingerprinting data matrices were subjected to non-supervised PCA to explore the analyzed honey samples distribution as well as the QC behavior. Then, the supervised classificatory PLS–DA method was applied according to the honey botanical varieties and geographical production regions. For PCA, the X-data matrix consisted of the non-targeted LC–HRMS fingerprint for each honey and QC sample, i.e., the ion signal intensity values as a function of *m*/*z* and retention time. In the case of PLS–DA, the same X-data matrix as PCA but without QCs was used, while the Y-data matrix defined the sample classes (i.e., the honey botanical variety or the honey geographical production region, depending on the case). The number of latent variables (LVs) required to obtain the PLS–DA models was estimated from the first relevant minimum of the cross-validation (CV) error from the Venetian blind approach when a matrix with more than 20 samples was used, or from leave-one-out CV when the matrices contained less than 20 samples.

## 4. Conclusions

A non-targeted LC–HRMS (Orbitrap) fingerprinting strategy was proposed for the characterization and classification of Spanish honey samples based on both the botanical varieties and geographical production regions. The proposed LC–HRMS chromatographic fingerprints were accomplished after a simple honey sample treatment consisting of dissolution in water and a 1:1 dilution with methanol followed by a C18 reversed-phase chromatographic separation using a universal gradient elution program.

The exploratory PCA and classificatory PLS–DA results from the LC–HRMS fingerprints demonstrated a superior descriptive performance in comparison to previous reported non-targeted HPLC–UV fingerprints, with the method being able to discriminate the Spanish honey samples based on their botanical varieties. The blossom honey samples were clustered into the five groups under study (BL, EU, HE, RO and TH) with only partial overlapping between the BL and RO samples, although their full separation was accomplished by paired PLS–DA. Highly satisfactory cross-validated multiclass predictions were generally attained, with sensitivity and specificity values higher than 96.2%, with the only exception of the BL and RO specificity values (57.1% and 79.6%, respectively). Low classification errors (below 1.7%) were also observed, except for BL and RO, which increased up to 21.4%. In the case of the honeydew honey samples, the three botanical varieties (FO, HO and MO honeys) were discriminated from the non-targeted LC–HRMS fingerprints, a fact that was not accomplished when using non-targeted HPLC–UV fingerprints, with sensitivity and specificity values of 100% and a 100% classification rate for the three classes.

In addition, the proposed non-targeted LC–HRMS fingerprints resulted in excellent chemical descriptors for honey classification according to three Spanish climatic geographical regions (CSE, LIR and MSR), a classification that was not previously accomplished with HPLC–UV fingerprints. The PLS–DA results were highly satisfactory, with good cross-validated multiclass prediction errors (sensitivity and specificity values higher than 87.1% and classification errors below 10.5%).

In general, the results attained in the present work demonstrated the superior performance of non-targeted LC–HRMS fingerprints for the classification and authentication of Spanish honey samples based on both the botanical varieties and climatic regions.

## Figures and Tables

**Table 1:** Multiclass predictions by cross-validation for the set of blossom-honey samples using three LVs. BL: orange/lemon blossom; RO: rosemary; EU: eucalyptus; TH: thyme, and HE: heather.

Sample Class Variety	Sensitivity (%)	Specificity (%)	Classification Error (%)
BL	100	57.1	21.4
RO	100	98.4	0.8
EU	100	96.6	1.7
TH	96.2	79.6	12.7
HE	100	100	0

**Table 2:** Multiclass predictions by cross-validation for the analyzed honey samples according to their geographical climatic production region using two LVs. CSR: Cantabrian Sea region; LIR: landlocked inland region; and MSR: Mediterranean Sea region.

Sample Class Variety	Sensitivity (%)	Specificity (%)	Classification Error (%)
CSR	94.7	99.0	3.1
LIR	92.4	98.2	4.7
MSR	91.9	87.1	10.5

**Table 3:** Universal gradient elution conditions.

Time (min)	Elution	% Acetonitrile	Flow Rate (µL/min)
0–5	Isocratic	3%	400
5–13	Linear gradient	3–95%	400
13–15	Isocratic	95%	400
15–15.5	Linear gradient	95–3%	400
15.5–19	Isocratic	3%	400

## Data Availability

Data is available upon request to the authors.

## Supplementary Materials

The following supporting information can be downloaded at: https://www.mdpi.com/article/10.3390/molecules27238357/s1, Figure S1: Non-targeted LC–HRMS (base peak chromatogram) fingerprints obtained for multifloral, eucalyptus, heather and forest honeys; Figure S2: Supervised PLS–DA score plots of LV1 vs. LV2 when using non-targeted LC–HRMS chromatographic fingerprints as honey chemical descriptors of orange/lemon blossom (BL) vs. rosemary (RO) blossom honeys (two LVs were used to build the model); Figure S3: Supervised paired PLS–DA score plots of LV1 vs. LV2 when using non-targeted LC–HRMS chromatographic fingerprints as honey chemical descriptors of climatic geographical production region. (**a**) LIR vs. MSR (three LVs were used to build the model), (**b**) CSR vs. MSR (two LVs were used to build the model) and (**c**) CSR vs. LIR (two LVs were used to build the model). CSR: Cantabrian Sea region; LIR: landlocked inland region; MSR: Mediterranean Sea region; Table S1: Multiclass predictions by cross-validation for the set of honeydew honey samples using three LVs. HO: holm oak; MO: mountain; and FO: forest; Table S2: Number of analyzed honey samples considering botanical varieties and geographical origins.

## Sample Availability

Samples are available upon request to the authors.
