# Supplementary material for: Characterization and Classification of Spanish Honey by Non-Targeted LC–HRMS (Orbitrap) Fingerprinting and Multivariate Chemometric Methods
Source: Molecules. 2022 Nov 30;27(23):8357. doi: 10.3390/molecules27238357 (PMC9740000; doi:10.3390/molecules27238357)
Supplement: Supplementary file 1 [file molecules-27-08357-s001.zip › molecules-2014241-supplementary.pdf]

Supplementary Material

Characterization and Classification of Spanish Honey by Non-targeted LC-HRMS (Orbitrap) Fingerprinting and Multivariate Chemometric Methods

Víctor García-Seval [1], Javier Saurina [1,2], Sònia Sentellas [1,2,3] and Oscar Núñez [1,2,3,*]

[1] Department of Chemical Engineering and Analytical Chemistry, University of Barcelona. Martí i Franquès 1-11, E08028, Barcelona, Spain
[2] Research Institute in Food Nutrition and Food Safety, University of Barcelona, Recinte Torribera, Av. Prat de la Riba 171, Edifici de Recerca (Gaudí), Santa Coloma de Gramenet, E08921 Barcelona, Spain.
[3] Serra Húnter Fellow, Generalitat de Catalunya, Via Laietana 2, E-08003 Barcelona, Spain.
* Correspondence: O.N.: oscar.nunez@ub.edu

Table S1. Multiclass predictions by cross-validation for the set of honeydew-honey samples using 3 LVs. HO: Holm oak; MO: Mountain; and FO: Forest.

Sample class variety	Sensitivity (%)	Specificity (%)	Classification Error (%)
HO	100	100	0
MO	100	100	0
FO	100	100	0

Table S2. Number of analyzed honey samples considering botanical varieties and geographical origins.

**Honey**	**Botanical variety**	**Number of samples**	**Geographical origin**	**Number of samples**
Blossom-honeys	Orange/Lemon Blossom (BL)	12	Aragon	1
			Balearic Islands	2
			Cantabria	1
			Castile and Leon	2
			Catalonia	3
			Extremadura	1
			Spain[a]	2
	Eucalyptus (EU)	13	Andalusia	1
			Asturias	2
			Cantabria	1
			Castile and Leon	2
			Catalonia	1
			Extremadura	4
			Navarre	1
			Spain and others[a]	1
	Rosemary (RO)	26	Andalusia	1
			Aragon	4
			Asturias	1
			Balearic Islands	4
			Castile and Leon	1
			Cantabria	2
			Catalonia	6
			Extremadura	4
			Navarre	1
			Spain[a]	2
	Thyme (TH)	7	Castile and Leon	1
			Castile La Mancha	2
			Catalonia	1
			Extremadura	2
			Spain[a]	1
	Heather (HE)	18	Asturias	2
			Basque Country	1
			Cantabria	3
			Castile and Leon	5
			Catalonia	2
			Extremadura	5
Honeydew-honeys	Mountain (MO)	6	Asturias	2
			Castile and Leon	1
			Castile La Mancha	1
			Catalonia	2
	Forest (FO)	10	Balearic Islands	2
			Cantabria	2
			Castile and Leon	1
			Catalonia	1
			Spain[a]	4
	Holm Oak (HO)	10	Aragon	2
			Castile and Leon	2
			Extremadura	6
Other honeys	Multifloral (MF)	34	Asturias	1
			Balearic Islands	4
			Cantabria	1
			Castile and Leon	7
			Castile La Mancha	2
			Catalonia	8
			Extremadura	4
			Navarre	4
			Spain[a]	1
			Spain and others[a]	2

[a] Spain: Honeys produced in Spain but geographical region not specified; Spain and others: Honeys that include mixtures of honey produced in Spain and other countries such as Uruguay, Cuba, Mexico, Romania or Ukraine

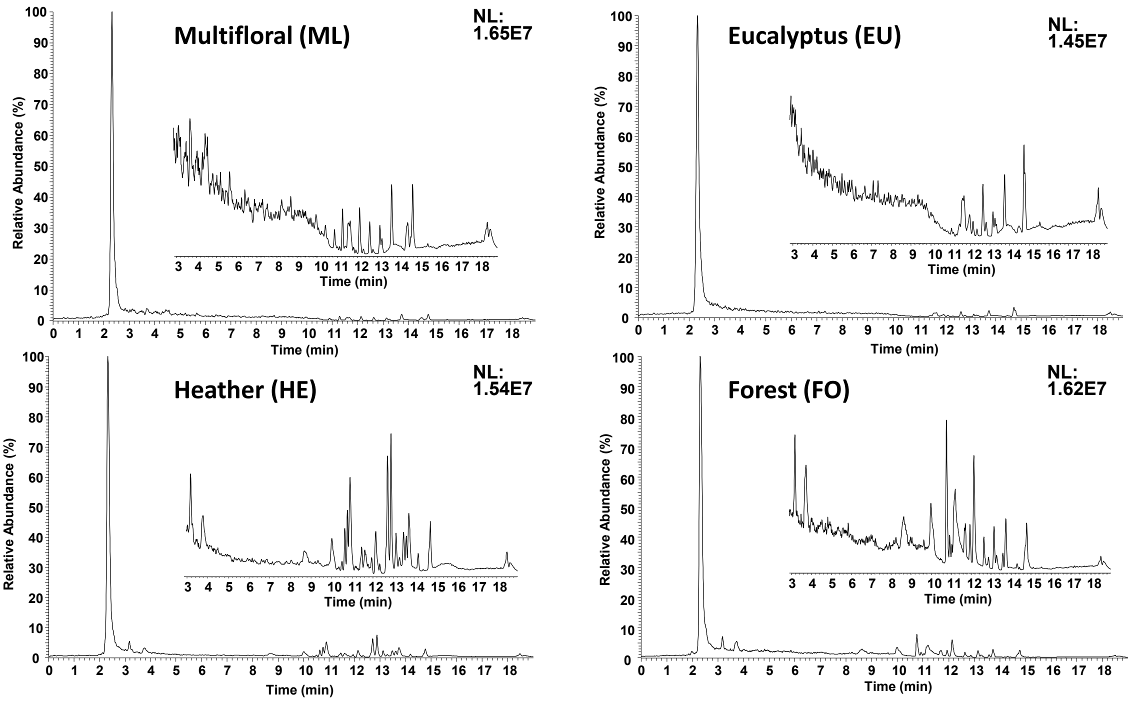

Figure S1. Non-targeted LC-HRMS (base peak chromatogram) fingerprints obtained for multifloral, eucalyptus, heather, and forest honeys.

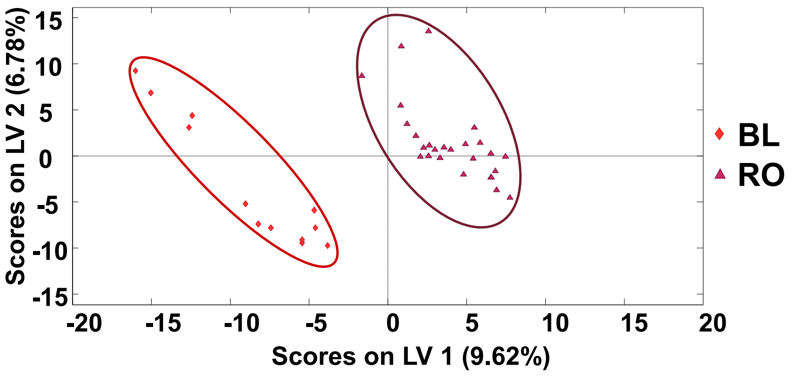

Figure S2. Supervised PLS-DA score plots of LV1 *vs*. LV2 when using non-targeted LC-HRMS fingerprints as honey chemical descriptors of orange/lemon blossom (BL) vs rosemary (RO) blossom honeys (2 LVs were used to build the model).

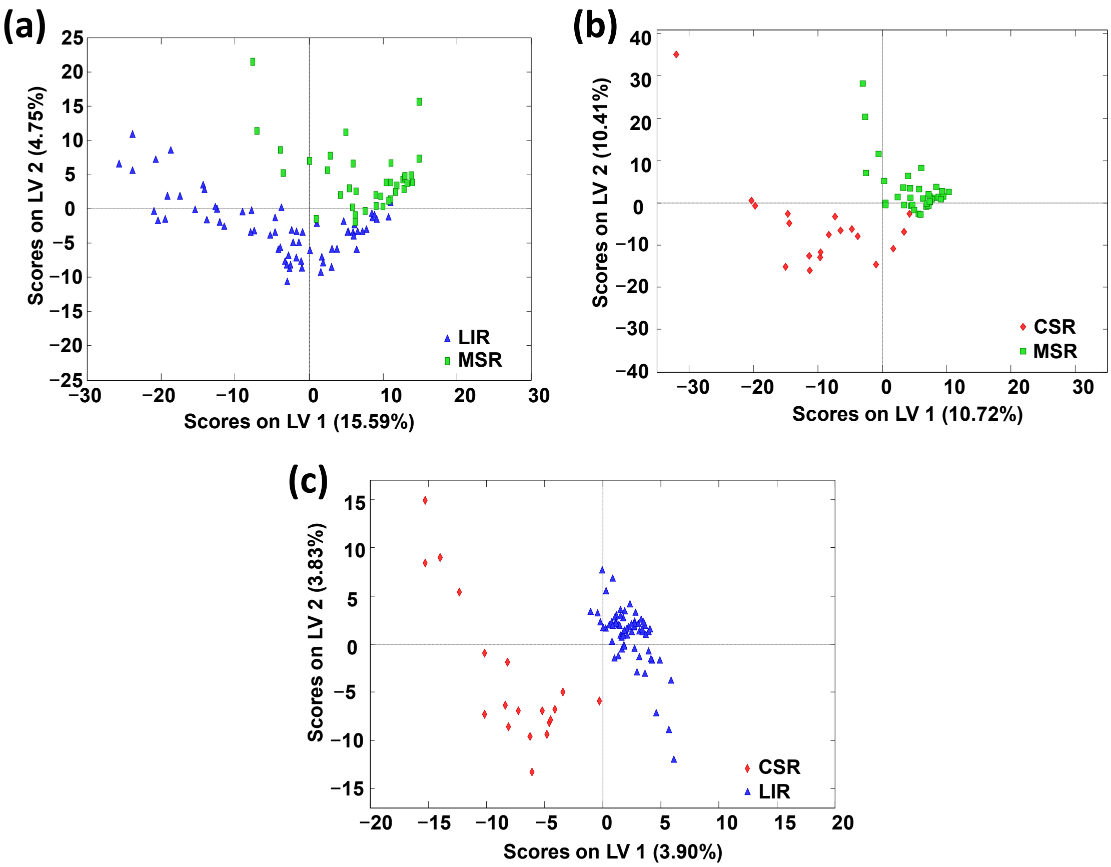

Figure S3. Paired PLS-DA score plots of LV1 vs. LV2 when using non-targeted LC-HRMS fingerprints as honey chemical descriptors of climatic geographical production region. (a) LIR *vs*. MSR (3 LVs were used to build the model), (b) CSR *vs*. MSR (2 LVs were used to build the model), and (c) CSR *vs*. LIR (2 LVs were used to build the model). CSR: Cantabrian Sea region; LIR: Landlock Inland region; MSR: Mediterranean Sea region.
